# Supplementary material for: Fine Mapping and Identification of BnaC06.FtsH1, a Lethal Gene That Regulates the PSII Repair Cycle in Brassica napus
Source: Int J Mol Sci. 2021 Feb 19;22(4):2087. doi: 10.3390/ijms22042087 (PMC7923215; doi:10.3390/ijms22042087)
Supplement: Supplementary file 1 [file ijms-22-02087-s001.pdf]

Figure S1. Chlorophyll a/b ratio and carotenoid/chlorophyll ratio in 7-521Y and 7-521G.

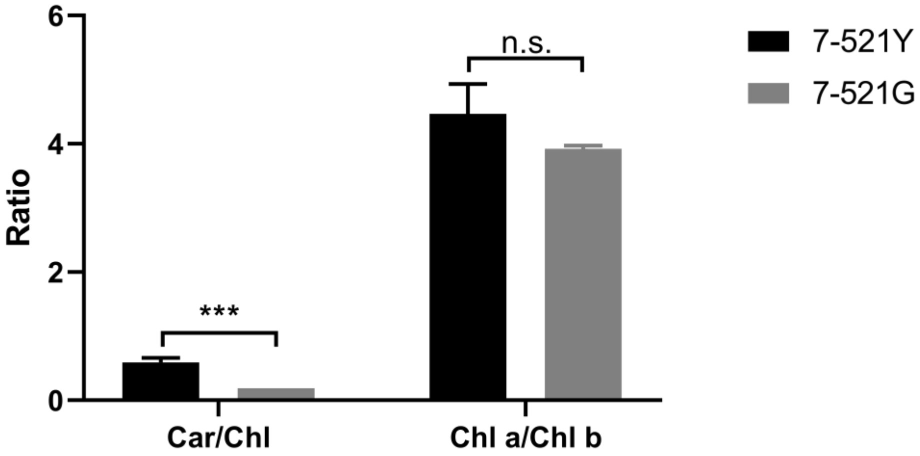

The significant differences were identified using a Student's t-test: n.s., not significant; asterisks indicate a significant difference: ***P < 0.001.

Figure S2. Transmission electron microscope (TEM) micrographs of the cotyledons from 7-521G and the mutant 7-521Y.

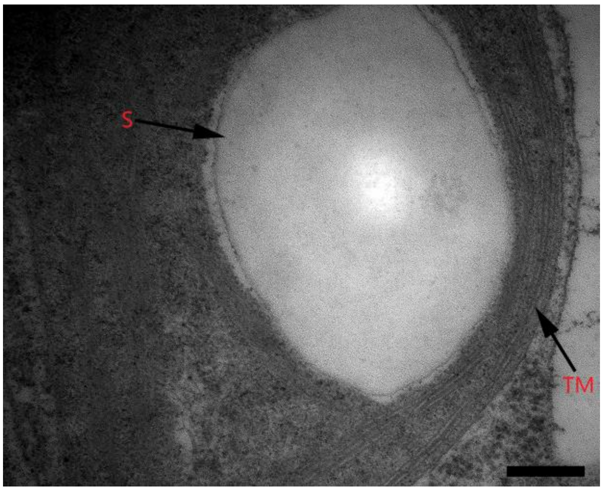

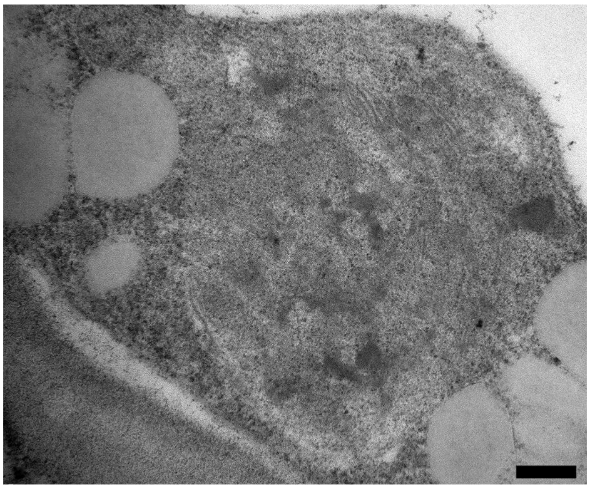

7-521G **7-521Y**

Stacked thylakoid membranes were observed in 7-521G (left), but were not observed in 7-521Y (right). S: starch grains, TM: thylakoid membrane. Bars: 300 nm.

Figure S3. Genotype of the three recombinants between S481 and S342 in the 15167 etiolated individuals.

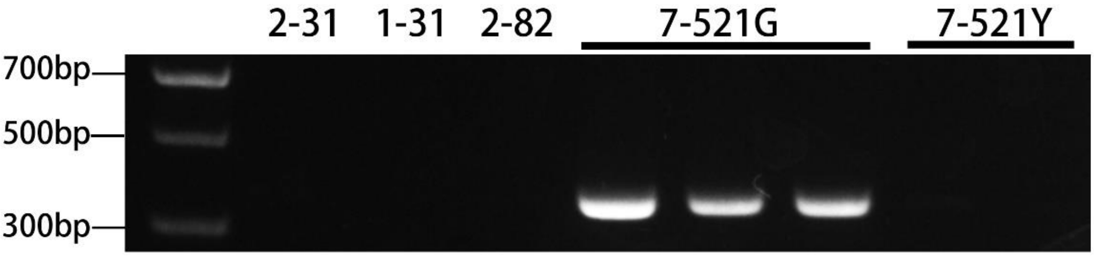

Detected using the Scar marker on a 2% agarose gel. The final three recombinants were 2-31, 1-31, 2-82, using 7-521Y as negative control and 7-521G as positive control.

Figure S4. Polymorphic markers on chrA07, ZG1-12 and ZY1-12 in F_3-1 population were used as templates

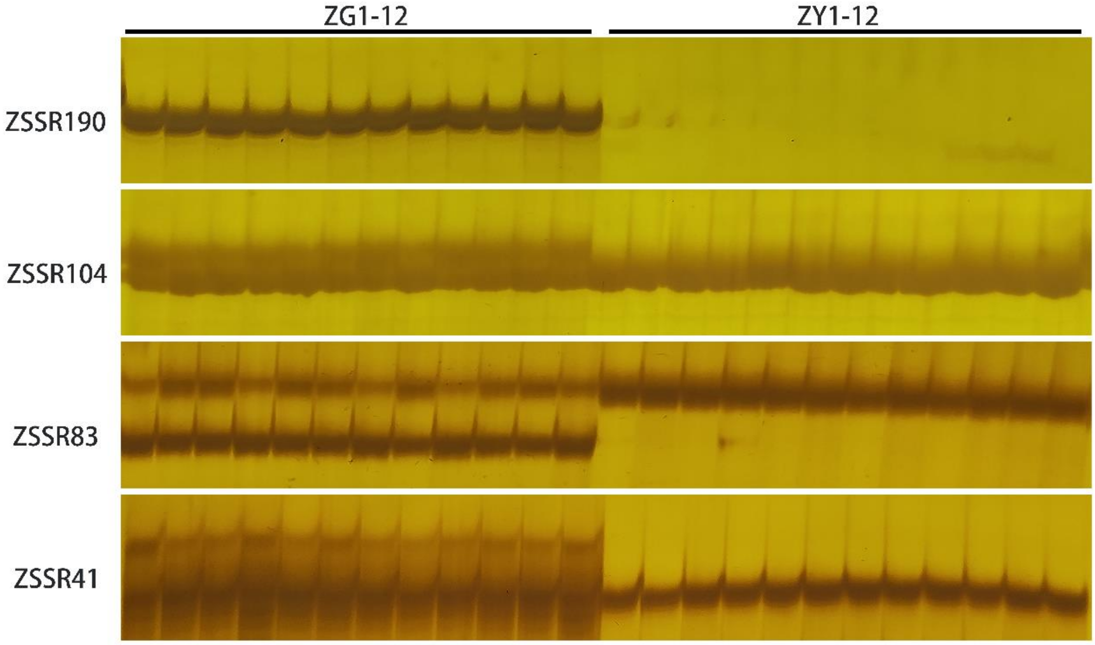

Multiple polymorphic markers were found in the F_3-1 segregating population. Normal phenotypes: ZG1-12, lethal phenotypes: ZY1-12.

Figure S5. The flow diagram of population development

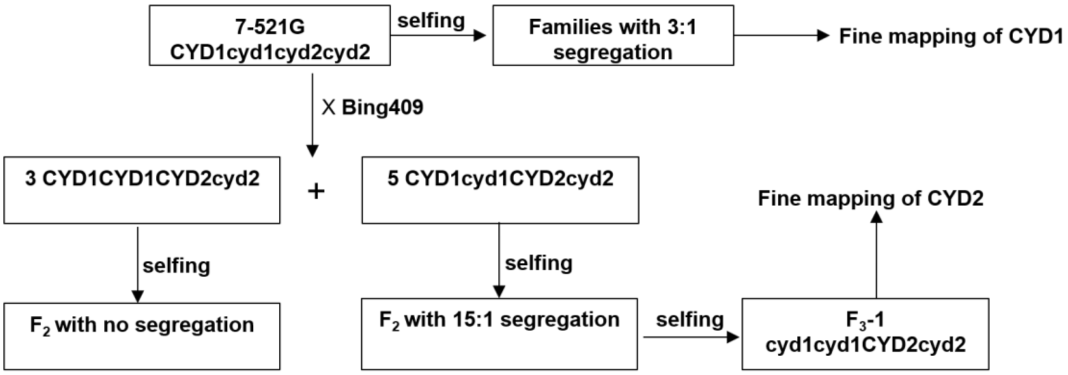

Table S1. Genetic analyses of the trait in F_2 Progenies

Lines	Green seedlings	Etiolated seedlings	Expected ratio	Chi-square value
140-3	190	9	15:1	0.723
140-30	157	8	15:1	0.336
140-54	140	8	15:1	0.074
140-73	103	6	15:1	0.014
140-86	204	8	15:1	1.848

Chi-square test, $\chi^2_{0.05,1}$= 3.84.

Table S2. Primers used in this study

**Primer**	**Sequences（5′ to 3′）**	**purpose**
S445F	ATCCAAAGCATCCTCTTCAACT	background
S445R	CTGGGAAAGTAATGGAGGGTAT	detection
S474F	TGCATGTGGTTGTTTTGG	background
S474R	CAGCTCGTGTACGTTTAGATTTC	detection
S342F	GGTTGATTCCAGTCGATTTGTT	background
S342R	ATGATCCAAAGCATAGCAAGC	detection
S349F	CAGCTTTCTCTCTTTGCCTCTC	background
S349R	TCCAGTGAAGGTGGAGCTTAAT	detection
M13-47F	CGCCAGGGTTTTCCCAGTCACGAC	transgene detection
A3-YXJC-11R	GCACCTATATCCATCTTCTTTGG	
In25F	CTCAATTATACTCGGACGAA	gene mapping
In25R	CCACCGAAAATCACCATAATA	
S312F	GGAGGAGGGAGGAAATCAATC	gene mapping
S312R	CACACACATACCAATCATCAAACC	
S361F	TGCATGTTGTGAGGTGTATGG	gene mapping
S361R	GCTGATATGATCCAATTTTCCC	
S394F	CCTTTGTGACTATCCTTCCTGC	gene mapping
S394R	CCCTTTTGGCTTTTGTCCTT	
S433F	ACAGGTCTACTAAGAGGCACGG	gene mapping
S433R	CAAAAGTGTCATGCTTCAAACG	
S439F	TCCCGAGAAGTCCACCTACTTA	gene mapping
S439R	GTGGGATGAAGAGAGAGAGGG	
S454F	GCAGTAGCTTTTCAACGATTCC	gene mapping
S454R	CCAGCTCGTAAACTATTCAGCG	
S457F	CGACCAATCAACAACTCCAA	gene mapping
S457R	CACCATGTGTTCTCCATCAAAT	
S466F	AGCATGTGGAGAGTAAGGGTGT	gene mapping
S466R	TCTGTAATGGCTGCTGATATGG	
S474F	TGCATGTGGTTGTTTTGG	gene mapping
S474R	CAGCTCGTGTACGTTTAGATTTC	
S481F	ATGCTGGTTTCAGGTCAAAT	gene mapping

S481R	TCCCCTCCCTCAAGTCATA	
ZSSR41F	GTTTGTTCCTACTTCATGCCG	gene mapping
ZSSR41R	CTTAAAAGCCAAATCAAGGGG	
ZSSR83F	TATTCGTGGGACACATTCTTGA	gene mapping
ZSSR83R	AGATGCTTAAATCGTAGCTGTGC	
ZSSR104F	ACTTAGCATCAACATCGCAAAC	gene mapping
ZSSR104R	TGGTAATGAGAGAGGACATGGA	
ZSSR190F	GGAGAGAGCAGAGAACGAAATC	gene mapping
ZSSR190R	GGCACTGGTACAGCCTAAAGAG	
FtsH1-C6GFL-F	CTCCGAACTTGCAAAGGGTTG	gene mapping
FtsH1-C6GFL-R	CATGGGTAATGGTCTAGTATAC	
FtsH1-C6-1F	GTTCGGTGGGCTTTTCTTGCTC	qRT-PCR
FtsH1-C6-1R	AACGCCTGTCTCGGGG	
D1-RT-1F	GGTTTGGTGTTTTGATGATCCC	qRT-PCR
D1-RT-1R	CCAATAGCTGCAGAAGTAGG	
ENGA -A5-RTF	TGGTGGTGTTATGACTGTTTCT	qRT-PCR
ENGA-A5-RTR	CATCTGACTCTTCAACAGCCGCT	
ENGA-C5-RTF	AGGAGGTGTCATGACTGTTTCC	qRT-PCR
ENGA-C5-RTR	CCTCGCAACAGCTGCCTCCCTA	
ENGA-A1-1F	GGACACCTATTGTTTATTCAT	qRT-PCR
ENGA-A1-1R	CTTGGTTTAAATGCAACAGCTAA	
ENGA-C1-1F	GGACACCTATTGTTTATTCAA	qRT-PCR
ENGA-C1-1R	CTTGGTTTAAATGCAACAGCTAG	
BnaENTH-F	GTTTAGACCCGTTGCTGCTC	qRT-PCR
BnaENTH-R	TTGTCCATCTCAGCCATTTG	
FtsH1-CZQC-3F	CTAGAGTCGACCTGCAGCAGGTCTTGAATACTTGCATACTG	vector construction
FtsH1-CZQC-3R	GCTTGCATGCCTGCAGCTTCATAGATTCACACTTGGAAGG	
C6SL-2F	AGCAGATCTATCGATTCTAGAATGGCTTCAATCTCGTTACGCT	vector construction
C6SL-2R	TCCTTTGCCCATGGCTCTAGAAGAAACATACAACTCAGCTTGGCC	
C6-P661+Sal1-F	GCGTCGTCGACTTATCGACTTGTTATTGTATAACATTTTCC	vector construction
C6-Pro+BamH1-R	TACTGGATCCTGTATGGGGACTAACTCTACTTCTTC	
